# Multi-scale supervised clustering-based feature selection for tumor classification and identification of biomarkers and targets on genomic data

**DOI:** 10.1186/s12864-020-07038-3

**Published:** 2020-09-22

**Authors:** Da Xu, Jialin Zhang, Hanxiao Xu, Yusen Zhang, Wei Chen, Rui Gao, Matthias Dehmer

**Affiliations:** 1 School of Mathematics and Statistics, Shandong University, Weihai, 264209 China; 2 School of Control Science and Engineering, Shandong University, Jinan, 250061 China; 3 Institute for Intelligent Production, Faculty for Management, University of Applied Sciences Upper Austria, Steyr Campus, Steyr, Austria; 4 College of Computer and Control Engineering, Nankai University, Tianjin, 300071 China; 5 Department of Mechatronics and Biomedical Computer Science, UMIT, Hall in Tyrol, Austria

**Keywords:** Feature selection, Machine learning, Classification, Biomarker, Therapeutic target, Clustering

## Abstract

**Background:**

The small number of samples and the curse of dimensionality hamper the better application of deep learning techniques for disease classification. Additionally, the performance of clustering-based feature selection algorithms is still far from being satisfactory due to their limitation in using unsupervised learning methods. To enhance interpretability and overcome this problem, we developed a novel feature selection algorithm. In the meantime, complex genomic data brought great challenges for the identification of biomarkers and therapeutic targets. The current some feature selection methods have the problem of low sensitivity and specificity in this field.

**Results:**

In this article, we designed a multi-scale clustering-based feature selection algorithm named MCBFS which simultaneously performs feature selection and model learning for genomic data analysis. The experimental results demonstrated that MCBFS is robust and effective by comparing it with seven benchmark and six state-of-the-art supervised methods on eight data sets. The visualization results and the statistical test showed that MCBFS can capture the informative genes and improve the interpretability and visualization of tumor gene expression and single-cell sequencing data. Additionally, we developed a general framework named McbfsNW using gene expression data and protein interaction data to identify robust biomarkers and therapeutic targets for diagnosis and therapy of diseases. The framework incorporates the MCBFS algorithm, network recognition ensemble algorithm and feature selection wrapper. McbfsNW has been applied to the lung adenocarcinoma (LUAD) data sets. The preliminary results demonstrated that higher prediction results can be attained by identified biomarkers on the independent LUAD data set, and we also structured a drug-target network which may be good for LUAD therapy.

**Conclusions:**

The proposed novel feature selection method is robust and effective for gene selection, classification, and visualization. The framework McbfsNW is practical and helpful for the identification of biomarkers and targets on genomic data. It is believed that the same methods and principles are extensible and applicable to other different kinds of data sets.

## Background

Genomic data, such as gene expression data, have been widely utilized to explore the mechanisms underlying a series of disorders [1]. It has the characteristics of imbalanced class distribution, a huge number of genes and a small number of samples. However, only a small subset of genes is suitable for tumor classification. To address these issues, some feature selection algorithms have recently been developed for identifying informative genes from genomic data of cancer [2–5].

Feature selection serves two purposes: to identify a subset of features that have the most discriminative information for the classification, to build rapid and robust predictive models and reduce the dimensionality of the data and to avoid over-fitting and improve classification accuracy [6, 7]; and to select relevant genes, and unravel the underlying biological mechanisms, or to be used as biomarkers or assess the efficacy of drugs [5, 8].

Based on the availability of labels in data, feature selection methods can be broadly categorized into unsupervised and supervised [9]. For example, Feng et al. recently developed a new supervised discriminative sparse PCA (SDSPCA) method for multiview biological data, which has been applied to cancer classification and informative gene selection [2]. Zhao et al. presented supervised and unsupervised spectral feature selection methods for handling high-dimensional data [10]. Supervised learning has been applied to single-cell transcription data to determine pathway activity and specific cell type. For example, Hu et al. described a novel methodology for identifying key markers in neocortical cells, using supervised learning [11]. A neural network-based approach can also be used to reduce the dimensions of single-cell RNA-seq data and predict cellular states and cell types [12].

In the literature, feature selection algorithms can be roughly grouped into three types: filter, wrapper, and embedded algorithms [13, 14]. The filter methods are independent of the classification algorithm, and they are faster than wrapper methods. Wrapper methods have higher learning capacity and search for optimal combinations of features. In general, filter methods can be considered the principal or auxiliary selection mechanisms. A better method is to use the univariate filter method to reduce the search space, and further apply wrapper or embedded feature selection methods.

In gene expression analyses, a powerful application of feature selection is to identify complex disease genes and biomarkers. Biomarkers can be used for disease early detection, prognosis, and assessment of drug efficacy [15]. Some feature selection methods have been presented for the identification of biomarkers [16, 17]. Embedding gene expression data into the network may obtain better interpretable gene sets and classification performance, biomarkers, or targets. Since disease development may involve pathways and genes in multiple biological processes, network-based approaches could better understand the deregulated molecular mechanisms of cancer development and progression [18]. In biological processes, certain genes and signaling pathways play central roles, which can be used as targets for disease therapy [19, 20]. Some network-based algorithms have been designed to select features or identify highly predictive biomarkers [1, 21].

First, we designed a multi-scale distance function. Then, using it, we proposed a new feature selection method called MCBFS that performs feature weighting and clustering in a supervised manner for finding the relevant features and removing the redundant features from the original feature set. In addition, we developed a general framework named McbfsNW to identify robust biomarkers and therapeutic targets for diagnosis and therapy of diseases. This mixed mechanism takes advantage of filter method, network analysis and wrapper method. First, candidate informative genes are selected from the original gene sets through MCBFS proposed in this work. Then, biomarkers and therapeutic targets are further identified by network analysis and more accurate wrappers.

## Results

### Datasets

To further assess the performance of MCBFS, the summary of ten publicly gene expression data sets used in the evaluation processes is tabulated in Table 1. Two-class cancer data sets and multi-class cancer data sets were used to compare the MCBFS method with other popular feature selection methods. Two-class cancer data set DLBCL, multi-class cancer data set SRBCT and two single-cell data sets were visualized through the MCBFS method and principal component analysis (PCA) to demonstrate our method is effective and widely applicable.

**Table 1:** Summary of ten gene expression data sets

Types	Data sets	Samples	Genes	Classes	References
Two-class cancer data sets	AMLALL	72	7129	2	[22]
	DLBCL	77	7129	2	[23]
	Gastric cancer	40	1519	2	[24]
	Colon Cancer	62	2000	2	[25]
Multi-class cancer data sets	Lymphoma	62	4026	3	[26]
	SRBCT	83	2308	4	[27]
	Brain-Tumor1	90	5920	5	[28]
	Lung-Cancer	203	12,600	5	[29]
Single-cell data sets	Pollen	249	14,805	11	[30]
	Usoskin	622	17,772	4	[31]

In order to evaluate the performance of the proposed biomarker and therapeutic target identification framework, we applied McbfsNW to lung adenocarcinoma (LUAD) data sets. Three original LUAD gene expression data sets (GSE10072, GSE7670 and GSE43458) were retrieved and downloaded from the Gene Expression Omnibus database (https://www.ncbi.nlm.nih.gov/geo/). To screen informative genes between the lung adenocarcinoma tissues and adjacent non-tumor tissues and balance the sample class sizes, we selected GSE10072 (107 samples), GSE7670 (54 samples from GSM185811 to GSM185864) and GSE43458 (70 samples from GSM1062805 to GSM1062874). In section 3.3.2, the combination of GSE10072 and GSE7670 was served as the training set, and GSE43458 was used as an independent test set to identify and verify biomarkers.

### The results of MCBFS

To obtain reliable results of MCBFS and make the results more representative, in this section, the experiment is divided into four parts. First, we plotted the MCBFS average classification error curves. Second, we compared different feature selection methods, including single distance method, seven benchmark and six state-of-the-art supervised feature selection methods. Third, the importance of informative genes selected was validated by visual assessment. Fourth, the differential expression of informative genes selected was analyzed by a two-sample t-test.

For the two-class cancer data sets, the average classification performance of the feature selection method was evaluated by several widely-used evaluation metrics, including accuracy (Acc), sensitivity (Sn), specificity (Sp) and F-score. The average classification performance of multi-class data sets was evaluated by Cohen’s Kappa coefficient (Kappa) [32] and Acc. After achieving a lower-dimensional representation of the data by feature selection, we adopted SVM (use RBF kernel) and kNN (k = 5) classifiers to classify the data, respectively. The cross-validation is a popular evaluation method and has been widely used in the field of bioinformatics and related studies [8, 16, 33]. We performed 10-fold cross-validation for 10 times to obtain a statistically reliable predictive performance. In this paper, the MCBFS method was tested on eight benchmark tumor data sets and compared with seven benchmark supervised feature selection methods [34], including Chi Square, Fisher Score, Information Gain, mRMR, Gini Index, Kruskal Wallis and Relief-F. In addition, to further evaluate the performance of MCBFS, we compared it with six state-of-the-art supervised feature selection methods, including supervised discriminative sparse PCA (SDSPCA) [2], infinite latent feature selection (ILFS) [14], Double Kernel-Based Clustering method for Gene Selection (DKBCGS) [3], Infinite Feature Selection (infFS) [6], Supervised Multi-Cluster Feature Selection (SMCFS) [9] and Spectral Feature Selection (SPEC) [10].

#### Classification error curves of MCBFS

**Fig. 1:**
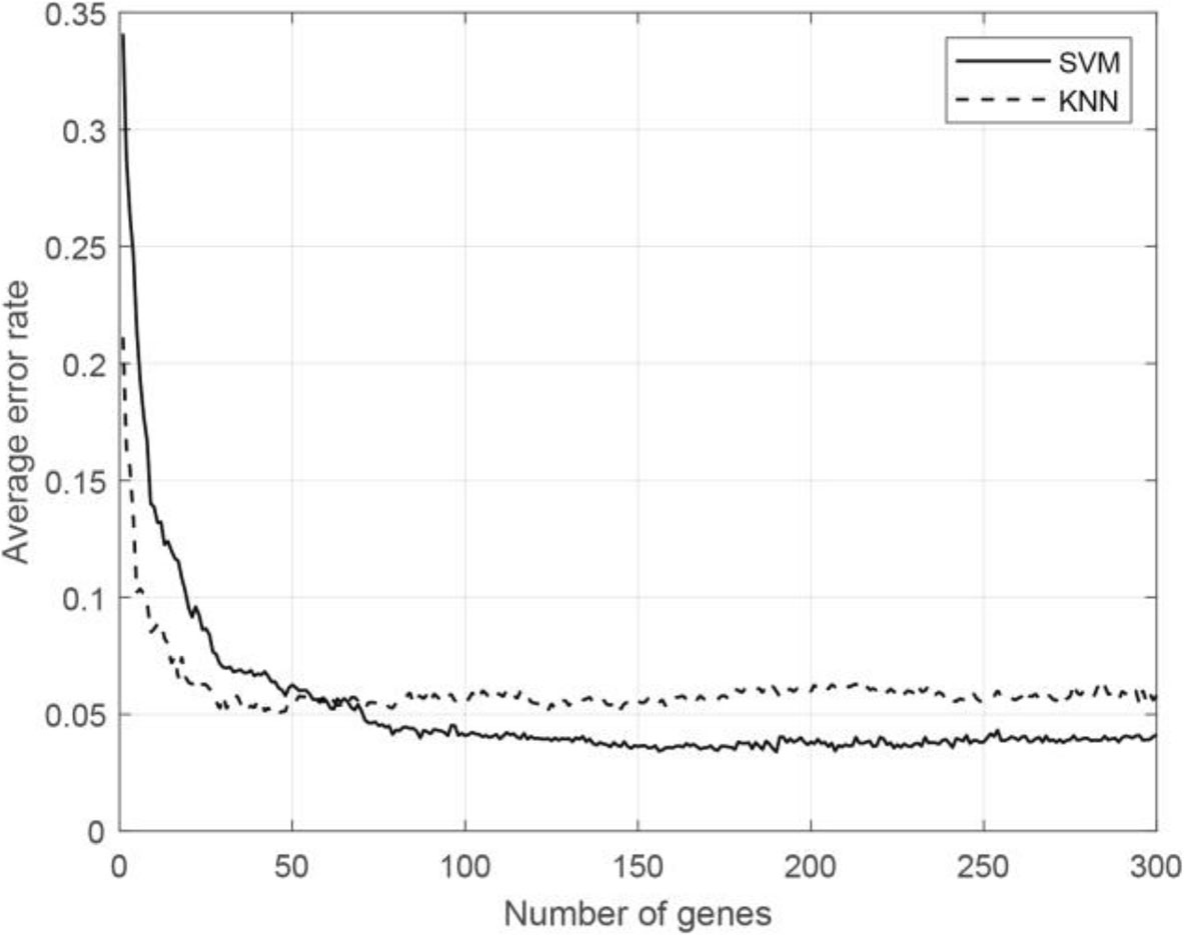
The relationship between the average classification error rates and the number of selected genes

The average classification error rates were obtained through 10-fold cross-validation with the kNN and SVM classifiers on eight data sets respectively. Figure 1 shows the relationship between the average classification error rate and the genes selected by the MCBFS method. From the figure, as the number of genes increases from 1 to 50, the predictable performance greatly improves. We set the range from 1 to 100 to find the best classification results. In general, most feature selection algorithms combine ranking genes with a specific classifier in the class prediction problems. From the figure, the kNN classifier has a better performance when fewer genes are retained. At the same time, the kNN classifier may be the better classifier for tumor classification with low-dimensional features [35]. In further work, to identify biomarkers on LUAD data sets by McbfsNW framework, the kNN classifier was applied in the wrapper.

#### Comparison of competitive methods

In this section, we compared MCBFS with different feature selection methods. The experiment is divided into three parts to obtain the performance of every method. The first part obtained the top 100 genes by using different methods, respectively. The second part used 10-fold cross-validation for 10 times to obtain the best feature set from retained the top 100 genes, respectively. In this part, the first ranked gene was used as the starting point of generating multiple gene subsets, which can save time for the generating subsets. The third part used the best feature subset and the 10-fold cross-validation for 10 times to obtain the average prediction performance of different methods.

**Fig. 2:**
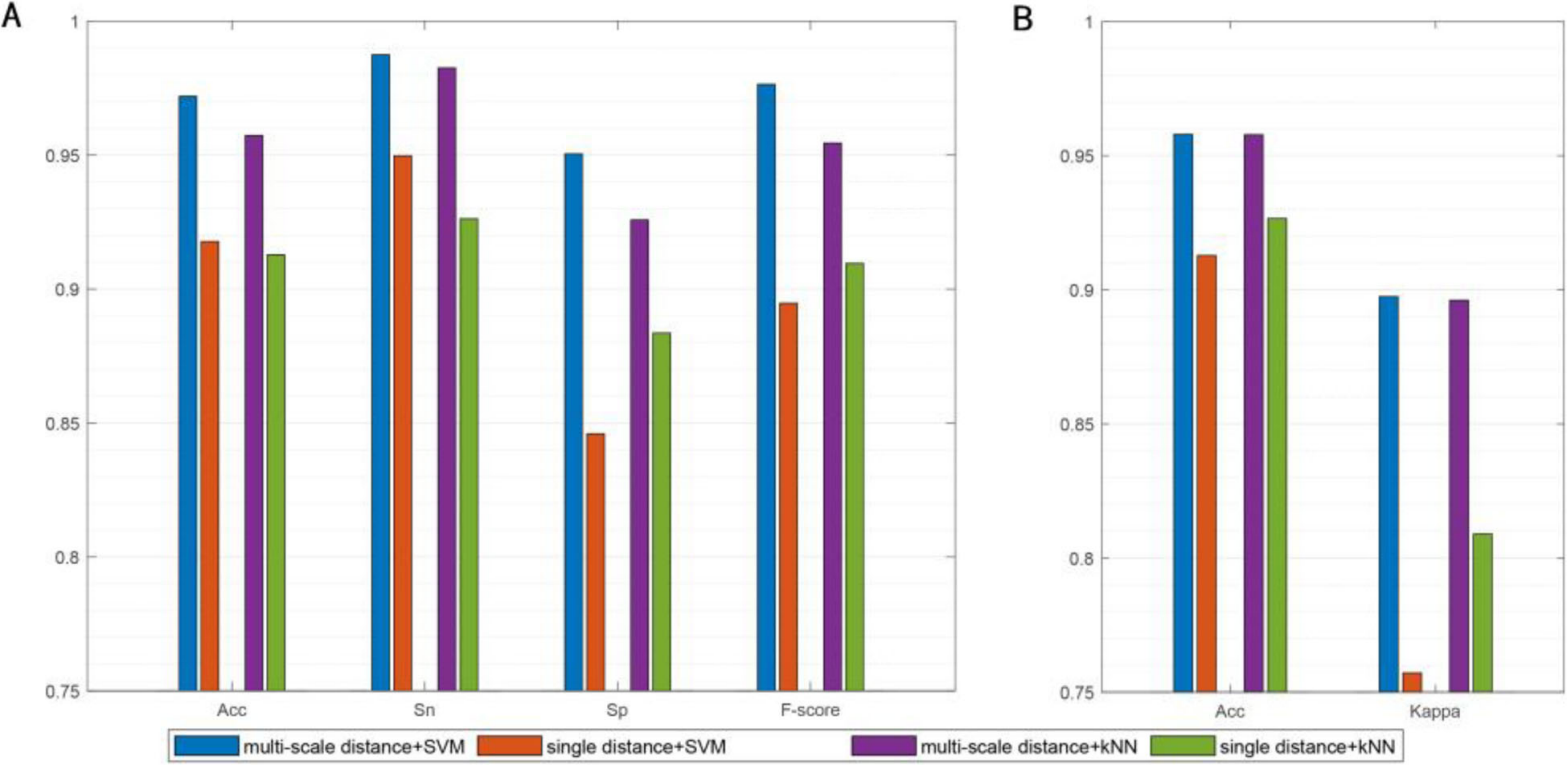
Comparison results of multi-scale distance method and single distance method. **a** The average results of four methods on four two-class cancer data sets. **b** The average results of four methods on four multi-class cancer data sets

To compare the performance of multi-scale distance method and single distance method, the average classification results were obtained on two-class cancer data sets and multi-class cancer data sets by SVM and kNN classifiers, respectively. In Fig. 2, we report the average performance of different distance methods for each type of data set. Figure 2a presents the average experimental results of four two-class data sets of two distance methods with SVM and kNN classifiers, respectively. From Fig. 2a, we can observe that multi-scale distance method achieves higher average results of four evaluation metrics. Figure 2b shows the average performance of four multi-class data sets on two distance methods with SVM and kNN classifiers, respectively. From Fig. 2b, we also can see a similar performance. It can be obtained that the performances of the multi-scale distance method yield better than the single distance method on two types of data sets. These results show the ability of the proposed multi-scale distance function and MCBFS.

**Fig. 3:**
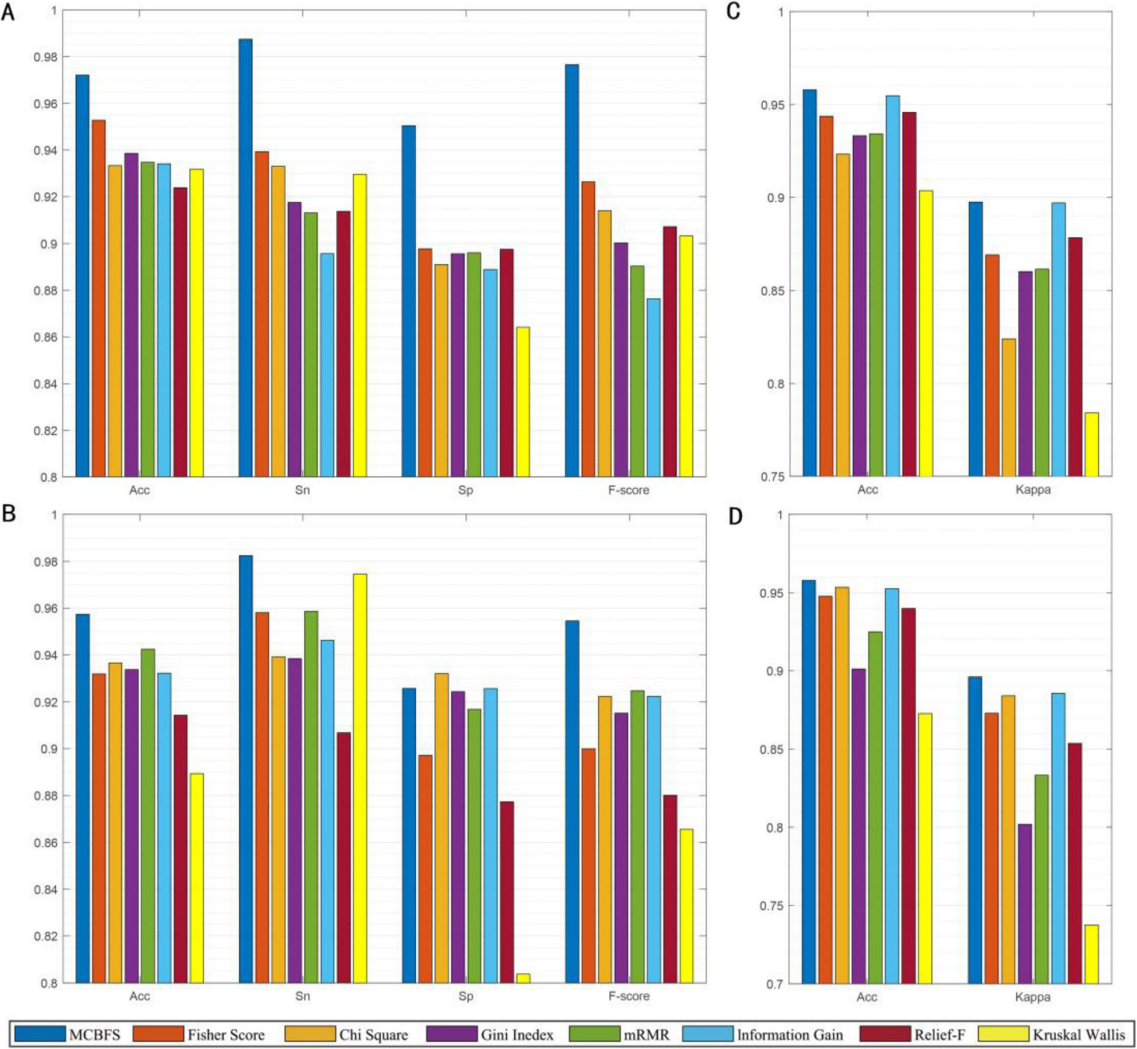
Comparison results of MCBFS and seven benchmark feature selections. **a** The average performance of four two-class data sets by different feature selection methods with SVM classifier. **b** The average performance of four two-class data sets by different feature selection methods with kNN classifier. **c** The average performance of four multi-class data sets by different feature selection methods with SVM classifier. **d** The average performance of four multi-class data sets by different feature selection methods with kNN classifier

To validate the classification performance of MCBFS, we compared it with seven popular supervised feature selection methods on eight benchmark micro-array data sets. In Fig. 3, we report the average performance of the different approaches for each type data set. Figure 3a and b present the average experimental results of four two-class data sets of eight feature selection methods with SVM and kNN classifiers, respectively. Figure 3c and d show the average performance of four multi-class data sets of eight feature selection methods with SVM and kNN classifiers, respectively. It is noteworthy that the MCBFS method can achieve better prediction performance than other methods, except is highly competitive to the Information Gain method with SVM classifier on the multi-class data sets.

**Fig. 4:**
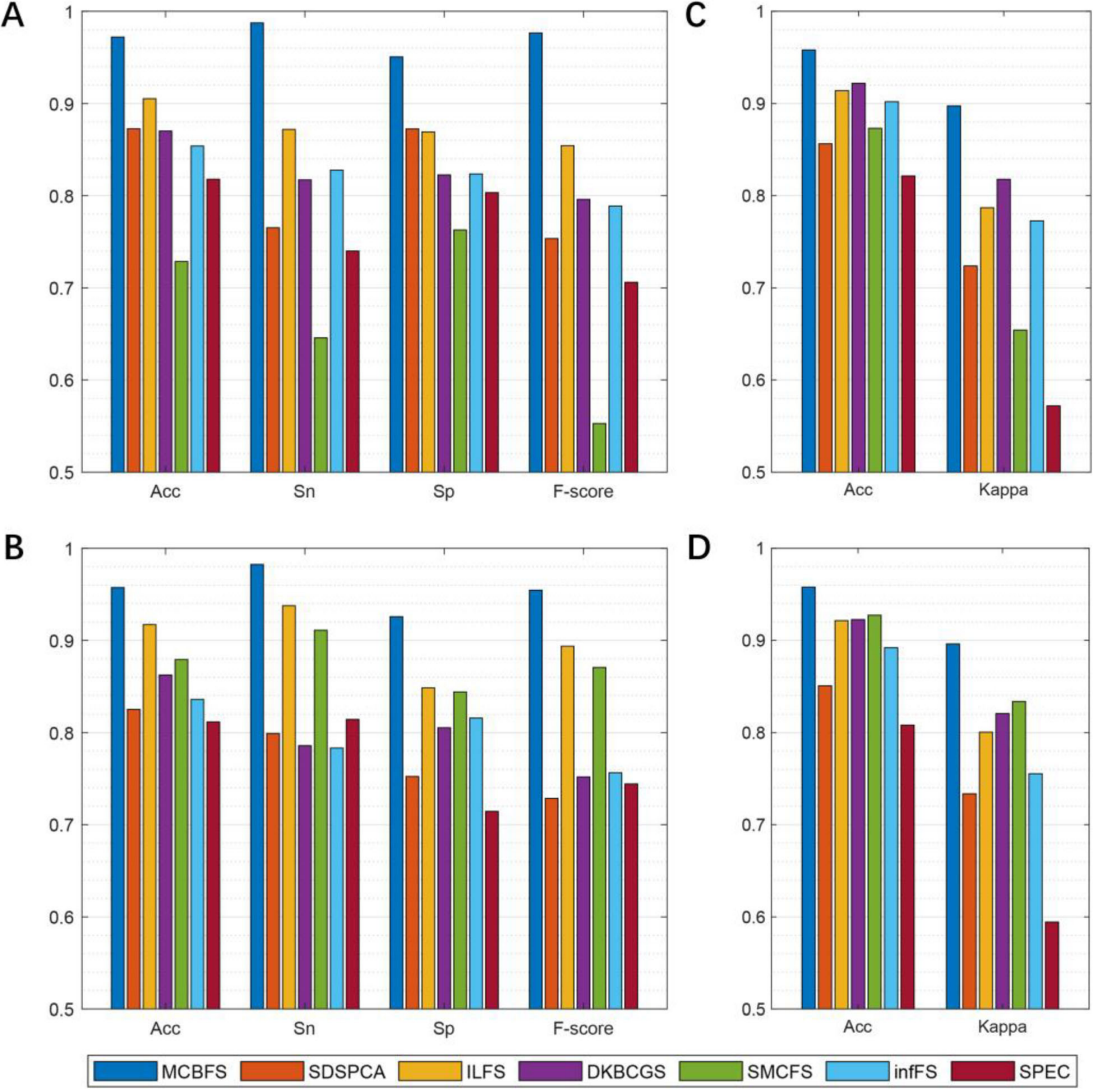
Comparison results of MCBFS and six state-of-the-art feature selections. **a** The average performance of four two-class data sets with SVM classifier. **b** The average performance of four two-class data sets with kNN classifier. **c** The average performance of four multi-class data sets with SVM classifier. **d** The average performance of four multi-class data sets with kNN classifier

In addition, we reproduced six state-of-the-art supervised feature selection methods and compared them with our method on two-class and multi-class data sets. The experimental process is similar to Fig. 3. The comparison results have been shown in Fig. 4. From Fig. 4, we can observe that MCBFS is superior to other methods. The experimental results can suggest that our method is a reliable and effective method for feature selection.

#### Visual assessment

**Fig. 5:**
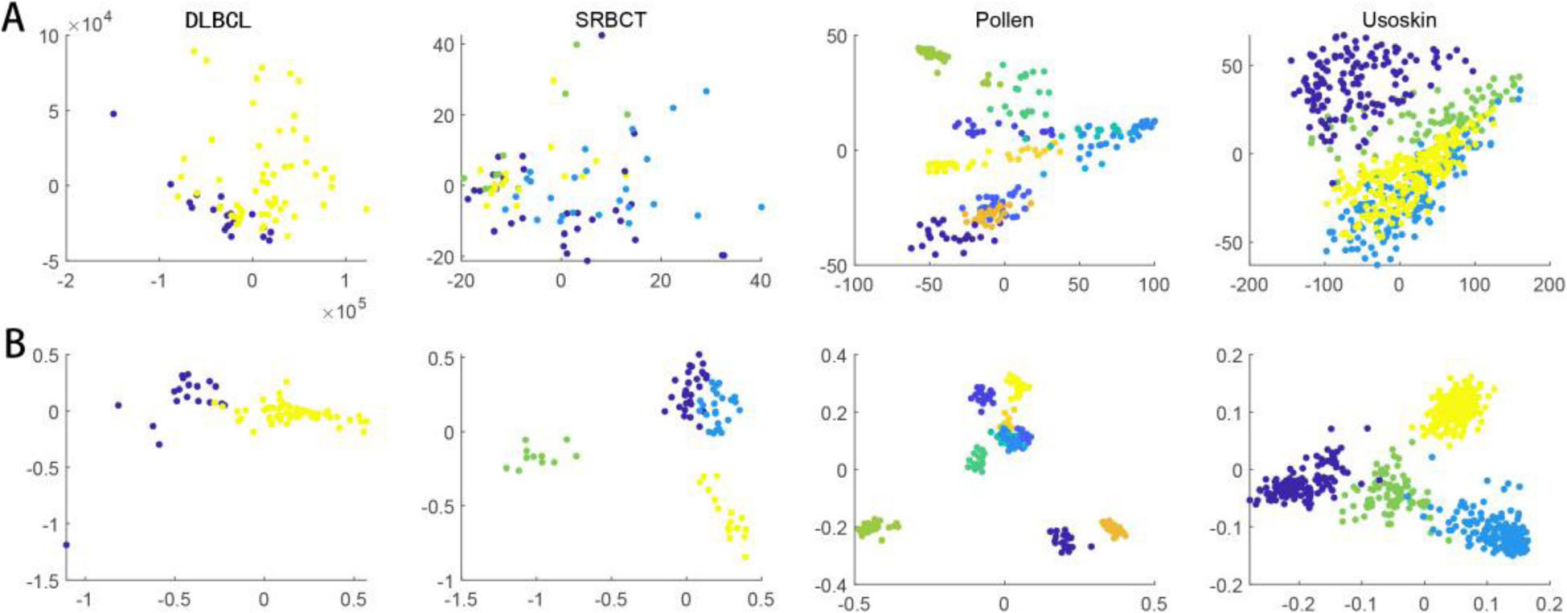
The sample distributions of four data sets are described by PCA**. a** PCA results of using all genes. **b** PCA results of using the top 100 genes

Given the sparsity and high dimensionality of gene expression data and single-cell sequencing data, the visualization of samples is used to validate the rationality of selected informative genes. Here, we displayed scatter plots with the two largest components of PCA. The visualization results of four data sets are shown in Fig. 5, respectively. For each data set, Fig. 5 a shows the PCA results of using all genes. The visualization results with the top 100 genes selected by MCBFS are shown in Fig. 5b. From Fig. 5b, we can see distinctly that using the top 100 genes obtained a better clustering result.

We can see tumor or cell populations clearly from Fig. 5b. More specifically, in Fig. 5b, the visualization results of the DLBCL and SRBCT data sets show that MCBFS can capture informative genes among tumor populations and improve the visualization and interpretability of tumor gene expression data. Single-cell RNA sequencing can enable us to discover new cell subtypes or types, and reveal the differences in gene expression among multiple cell populations [36]. In Fig. 5b, the visualization results of the Pollen and Usoskin data sets show that MCBFS is scalable, which can also capture informative genes among cell populations. MCBFS may be an effective tool for finding key markers from genomic data.

#### Statistical test

**Fig. 6:**
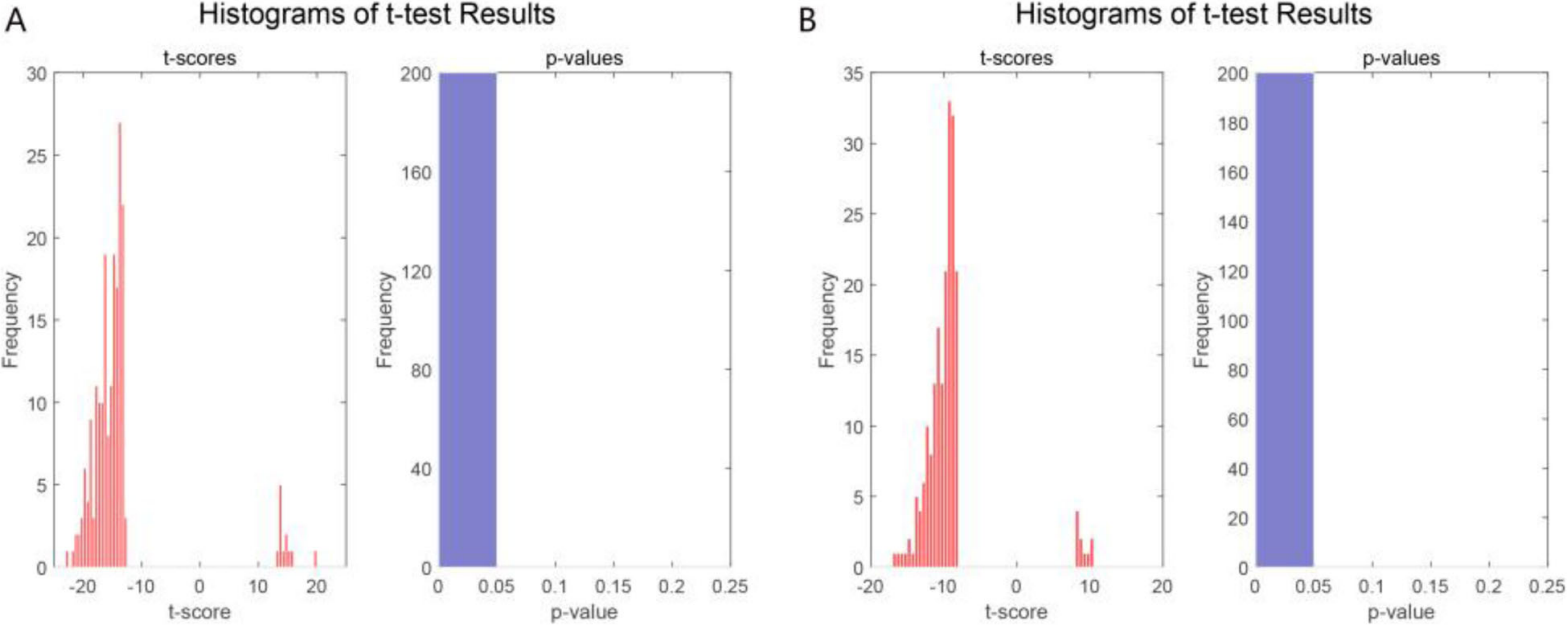
The t-test results of genes. **a** The t-test results of the top 200 genes selected by MCBFS in GSE10072. **b** The t-test results of the top 200 genes selected by MCBFS in GSE7670

Another important application area of feature selection is to detect the differentially expressed genes. To prove that the informative genes selected by the MCBFS method are differential expression and evaluate differential expression of genes in different phenotypes, the top 200 informative genes of GSE10072 and GSE7670 selected by the MCBFS method were analyzed by two-sample t-test respectively [37]. The results have been displayed by normal t-score quantile plots, histograms of t-score and *p*-value distribution in Figs. 6 and S1 (supplementary data). If the *p*-value of the gene is no more than 0.05, this gene will be considered a significant difference. The histogram of the t-score can give a sense of the density of the underlying distribution of selected genes. Figures 6 and S1 illustrate these informative genes are differentially expressed in the LUAD samples. These experimental results prove that MCBFS has a certain statistical significance and may be efficient in identifying differentially expressed genes.

### Application of McbfsNW on LUAD data

To evaluate the performance of McbfsNW framework for the identification of biomarkers and therapeutic targets, we applied it to the LUAD data sets. In lung cancer, there are two main pathological types: small cell lung cancer (SCLC) and non-small cell lung cancer (NSCLC). NSCLC accounts for approximately 85% of the total number of lung cancer cases [38]. It is worth noting that LUAD is one of the most important subtypes in NSCLC.

#### Identification of hub informative genes

We selected 200 informative genes by MCBFS in two independent micro-array data sets, GSE10072 and GSE7670, respectively. There was an overlap between two LUAD data sets: a total of 130 shared informative genes. As shown in Figure S2, the overlapping areas indicate shared informative genes. The complex protein-protein interaction network formed by proteins encoded through shared informative genes is shown in Figure S3, after eliminating disconnected nodes. Ten hub informative genes were screened by the network recognition ensemble algorithm, including PECAM1, CDH5, CAV1, CLDN5, SPP1, AGTR1, ANGPT1, FABP4, TEK and GJA4. They are shown in yellow in the network. There is mounting evidence that has reported these genes are significantly correlated with LUAD or NSCLC. The evidence of ten hub informative genes is tabulated in Table 2.

**Table 2:** Summary of ten hub informative genes

Gene name	Protein name	*P* value	Reference
TEK	Angiopoietin-1 receptor	8.90e-10	[39]
ANGPT1	Angiopoietin-1	4.30e-05	[40]
CAV1	Caveolin-1	4.90e-05	[41]
SPP1	Osteopontin Secreted phosphoprotein 1	0.0015	[42]
CDH5	Cadherin-5	0.0034	[43]
PECAM1	Platelet endothelial cell adhesion molecule	0.0036	[44]
CLDN5	Claudin-5	0.045	[45]
AGTR1	Type-1 angiotensin II receptor	0.054	[46]
GJA4	Gap junction alpha-4 protein	0.13	[47]
FABP4	Fatty acid-binding protein	0.25	[48]

**Fig. 7:**
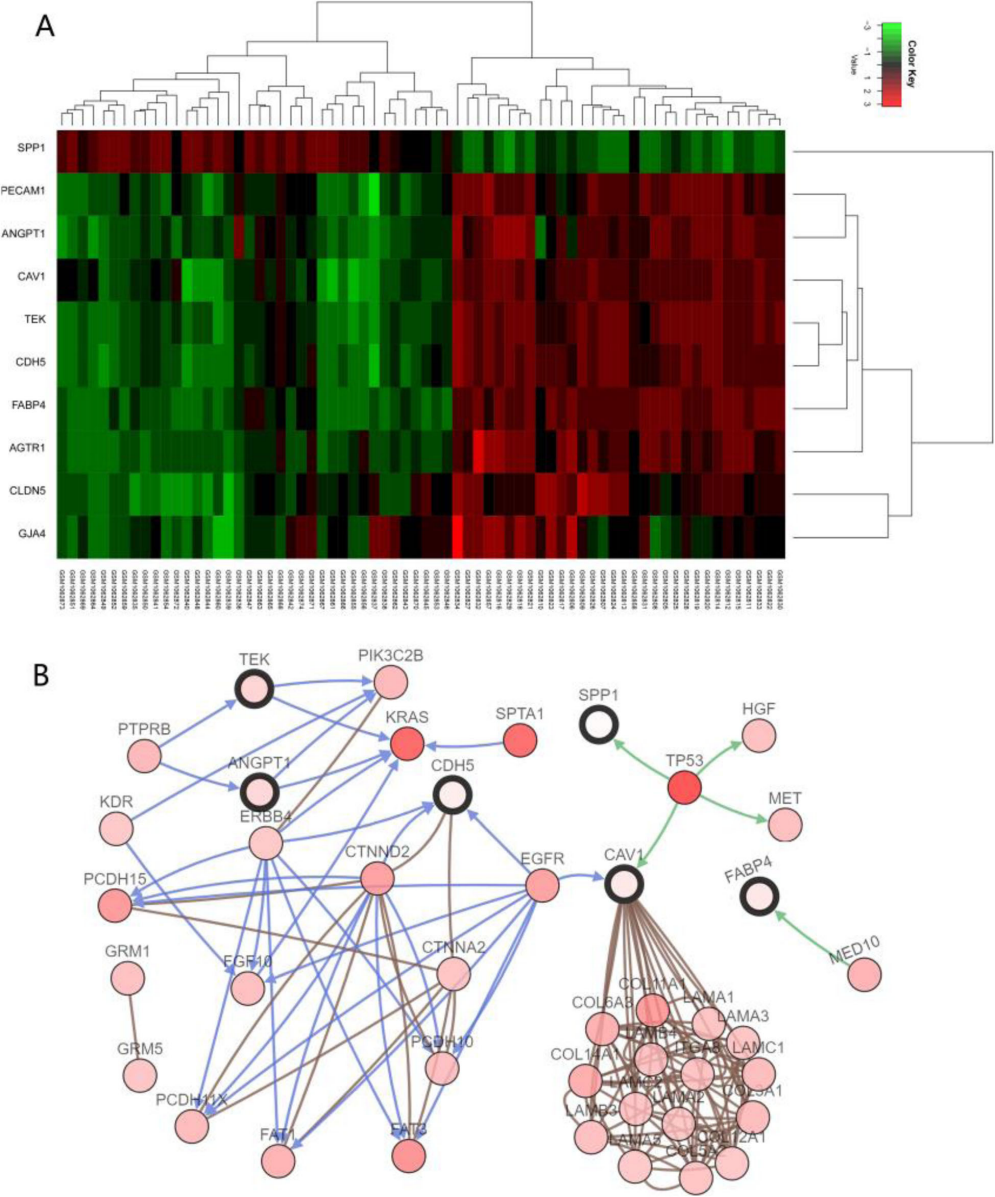
**a** The cluster heat map of 10 hub gene expressions. **b** Genetic alterations network of hub genes

Figure 7a displays the heat map of the 10 hub genes on the GSE43458 data set. It was generated by the R package “heatmap”. In the ten hub genes, nine low-expression genes are related to LUAD, including PECAM1, CDH5, CAV1, CLDN5, AGTR1, ANGPT1, FABP4, TEK and GJA4. In addition, there is a highly expressed gene SPP1. From the figure, we can see that data samples from different classes have distinctive expression profiles that lead to a reasonable classification performance.

Identification of major genetic changes leading to the inactivation of tumor suppressor genes and the activation of oncogenes has the potential to elucidate molecular mechanisms. We constructed a genetic alterations network with the 10 hub genes using the cBioPortal (http://www.cbioportal.org) [49]. Figure 7b demonstrates the relationship between the 10 hub genes and the other 50 most frequently altered neighbor genes (only CDH5, SPP1, CAV1, TEK, ANGPT1 and FABP4 have connection with these 50 genes).

From Fig. 7b, it is worth noting that (1) SPP1 and CAV1 are relevant to TP53. TP53 is associated with a variety of human cancers and encodes a tumor suppressor protein. The inactivation of TP53 is one of the most important genetic abnormalities in lung cancer. (2) In addition, CDH5 and CAV1 have a direct relationship with EGFR. EGFR is involved in the regulations of many oncogenic functions, such as cell differentiation, neovascularization, invasion, metastasis and survival. It is worth noting that almost all EGFR mutations occur in LUAD. (3) Beyond that, TEK and ANGPT1 are relevant to KRAS. The transforming protein of KRAS is implicated in various malignancies, including LUAD and colorectal carcinoma. The activating mutation of the KRAS oncogene is the most common oncogenic alteration in LUAD, which occurs in approximately 25–40% of cases. The details of TP53, EGFR and KRAS can be found in the lung cancer review paper [38, 50].

More importantly, these results show that the ten hub informative genes have possible biological relationships with the development and treatment of LUAD, which can provide novel insights for the pathogenesis of tumor. They can serve as candidate biomarkers or promising targets of LUAD.

#### Identification of biomarkers

Hub genes with *P* values no more than 0.05 were further screened as key genes in the survival analysis (Figure S4). We ordered 10 hub genes according to their *P* values in Table 2. Survival analysis suggested that seven genes were significantly related to the survival time of patients, including TEK, ANGPT1, CAV1, SPP1, CDH5, PECAM1 and CLDN5. They were screened as key genes.

**Fig. 8:**
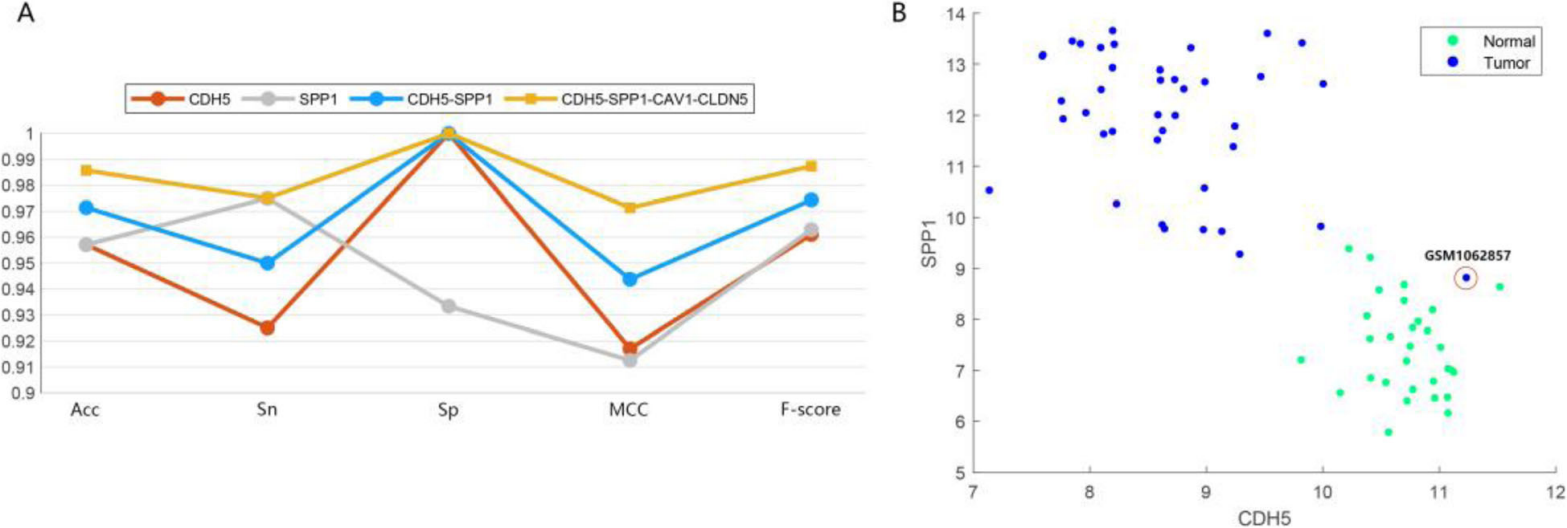
**a** The classification performances of typical combinations in the key genes. **b** Expression levels of two genes, SPP1 and CDH5, in LUAD 70 tissue samples

To further explore the possibility of some genes among the key genes as biomarkers, the combination of GSE10072 and GSE7670 was used as a training set, and another independent RNA-seq data set, GSE43458, was used as a testing set to determine the classification performances. Apart from Acc, Sn, Sp, and F-score evaluation metrics, we also used the Matthews correlation coefficient (MCC) for evaluation. The comparison results and typical combinations of key genes have been shown in Fig. 8a. The results show that the key genes can achieve very high classification performance. Specifically, the predictive accuracy of CDH5 and CAV1 is 95.71%, simultaneously. The predictive accuracy of the combination of CDH5 and CAV1 is 97.14%. The experimental results prove that obtained biomarkers can achieve higher prediction results and McbfsNW may be a useful tool for finding possible biomarkers from genomic data.

Using a small number of genes can achieve a good classification performance. To visualize the GSE43458 data set containing 70 samples, we plotted Fig. 8b by the expression levels of two genes (SPP1 and CDH5). As shown in Fig. 8b, most tumor samples and normal tissue samples are separated. This simple prediction rule with two genes can help medical doctors to make a simple pre-clinical diagnosis of the LUAD. A useful function of the visualization is to detect possible outliers. From Fig. 8b, we can see that sample GSM1062857 is abnormal and allocated to the normal group, which can be further studied in the future. The outlier is mainly due to the heterogeneity among the tumors of different patients [51]. CDH5 and SPP1 may be served as potential biomarkers in the early diagnosis of LUAD.

#### Drug-target network

**Fig. 9:**
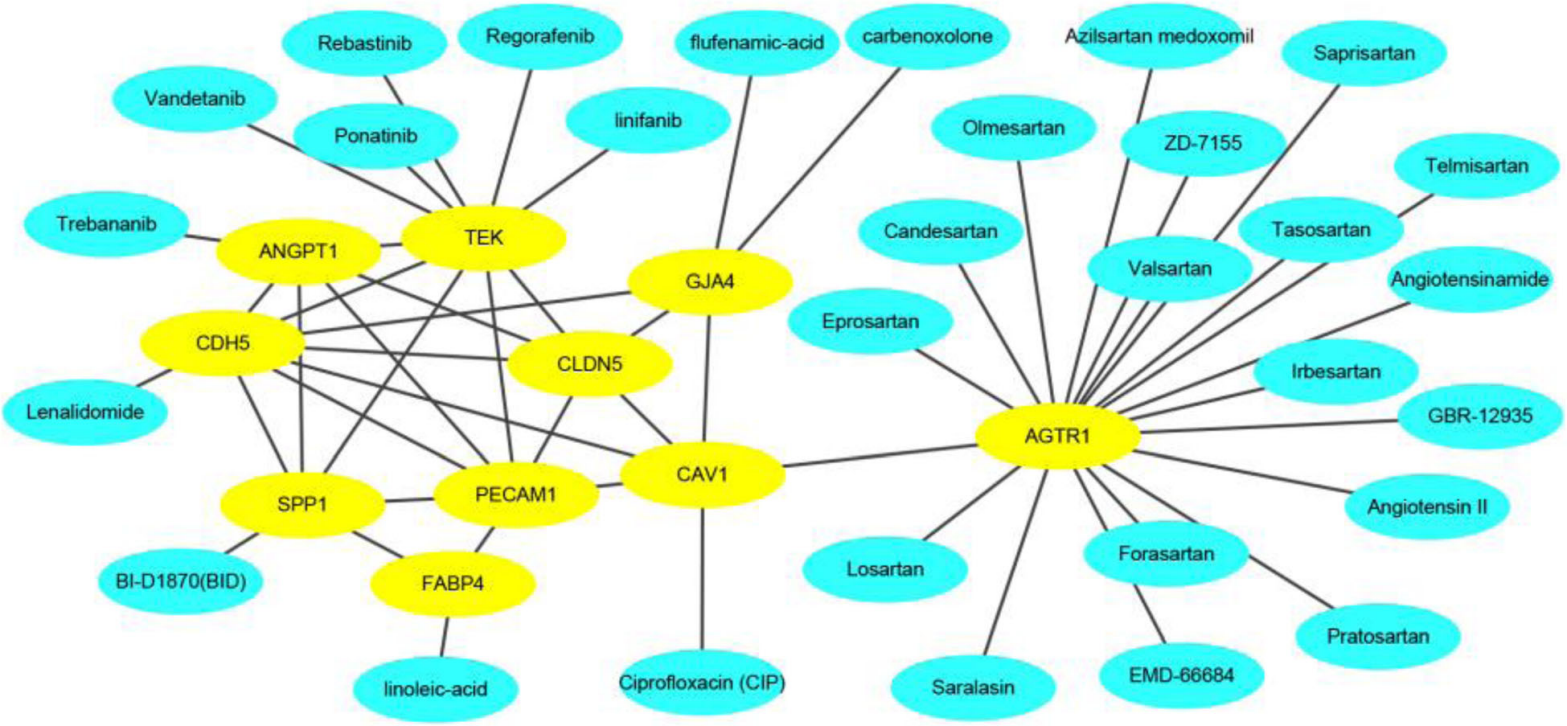
The integrative drug-target network

Given that 10 hub genes or proteins encoded by 10 hub genes may be promising targets of LUAD, we want to explore potential therapeutic drugs for effective treatment. We integrated three different aspects to find potential drugs, including the Connectivity Map L1000 platform [52] (https://clue.io), the cBioPortal and related literature. The drug-target network was plotted in Fig. 9. The yellow filled nodes represent targets in the drug-target network.

In this work, we pay more attention to the development and application of the algorithm. We verified the effectiveness of the algorithm in the proven literature. It is worth mentioning that many drugs have been proven to be effective for LUAD or NSCLC, including Vandetanib [53], Linfanib [54], Candesartan [55], Telmisartan [56], Lenalidomide [57], Regorafenib [58], Ponatinib [59], linoleic-acid [60], BI-D1870 (BID) [61] and Ciprofloxacin [62], and so on. More work needs to be performed to verify other drugs’ utility. Briefly, these targets and compounds provide a promising list for researchers or companies who are interested in the mechanisms of LUAD treatment.

The experimental results demonstrate that biomarkers identified by the McbfsNW framework can achieve a higher prediction performance for LUAD disease. There some therapeutic targets obtained by the McbfsNW framework have been proven to be effective for LUAD therapy. The framework may be a useful tool for finding possible biomarkers and therapeutic targets from genomic data.

## Discussion and conclusion

In this study, we proposed a multi-scale clustering-based feature selection method for gene expression data, MCBFS, which performs clustering and feature weighting in a supervised manner. In the algorithm, a multi-scale distance function designed by us was used as a dissimilarity measure. Based on the experimental results, MCBFS has significant advantages in terms of classification performance compared with 7 benchmark and 6 state-of-the-art feature selection algorithms. The visualization results and the statistical test show that MCBFS can capture informative genes among tumor populations or cell populations, which can improve the interpretability and visualization and of tumor gene expression data or single-cell data. The reasons for the effective performance of MCBFS are as follows: multi-scale distance function dissimilarity measure improves the performance of the algorithm; the adaptive distance changes at each iteration, which is suitable for learning the optimal weight of genes in the process of non-parametric clustering; furthermore, for a new data set, MCBFS does not require any parameter to be tuned manually.

Given that biomarkers should have higher specificity and sensitivity, we developed a general framework named McbfsNW, which uses gene expression and protein interaction data to identify biomarkers and therapeutic targets. The mixing mechanism of McbfsNW takes advantage of filter, network and wrapper. First, candidate informative genes were selected from the original gene sets by MCBFS. Then, biomarkers and therapeutic targets were further identified by the network recognition ensemble algorithm and a more accurate wrapper with exhaustive best subset search. To evaluate the performance of McbfsNW, we applied it to LUAD data sets. The experimental results showed that better prediction results can be achieved by identified biomarkers. Many drugs in the drug-target network were supported by published literature.

The MCBFS algorithm and McbfsNW framework are scalable and can also be applied to other genomic data for dimension reduction, identification of differentially expressed genes, sample classification or identification of biomarkers and therapeutic targets. Although MCBFS and McbfsNW have good performances for gene expression data, there are some limitations in this work. MCBFS is a method based on multi-scale, which may be time consuming. For the partial results of McbfsNW, due to the limitations of laboratory conditions, we can only verify them in the previous literature. If the laboratory conditions permit, we would very much like to further validate relevant findings in our later works. The visualization result of GSE43458 data set based on the expression levels of two genes can discover the outlier that does not satisfy the prediction rule. The outlier is mainly due to heterogeneity among the tumors of different patients, and the mutations of the abnormal patient are almost different from other patients in the founder cells of the tumor. We could further focus on and study these abnormal patients in the future.

It is conceivable that the same principles and methods can be applied to other types of genomic data, for example, DNA methylation data or copy number variation data, which play important roles in tumorigenesis. It could even be possible to integrate all these data into a unified model to better identify robust biomarkers and therapeutic targets. We believe that this work provides a refreshing view on the identification of biomarkers and therapeutic targets by feature selection and network analysis.

## Methods

**Fig. 10:**
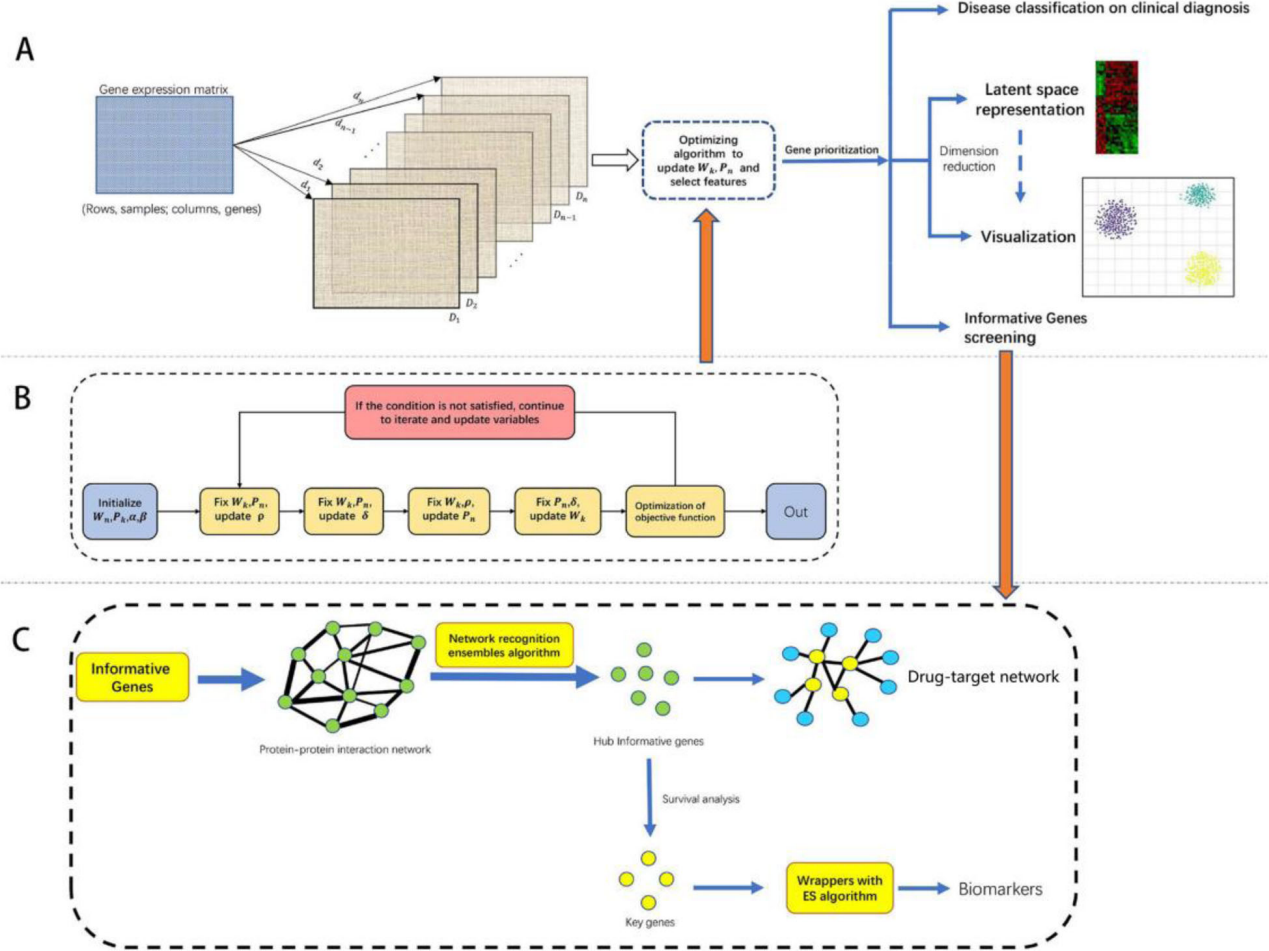
The flowchart of McbfsNW. **a** The workflow of the MCBFS algorithm. **b** The iterative process of the MCBFS algorithm. **c** The network analysis and wrapper of McbfsNW

In this paper, a novel feature selection method named MCBFS is proposed, which simultaneously performs model learning and feature selection for high-dimension data. The details of MCBFS are presented in Fig. 10a and b. In addition, we develop a general framework named McbfsNW to identify robust biomarkers and therapeutic targets for diagnosis and therapy of diseases, which incorporates feature selection with network analysis into pattern recognition in the biological process. The workflow of McbfsNW is shown in Fig. 10.

### Feature selection (MCBFS)

Gene expression data set *X* ∈ *R*^*s* ∗ *p*^ is comprised of *s* samples. Each sample is represented by a row vector *X*_*j*_ ∈ *R*^*p*^, and labeled by *y*_*j*_ ∈ *Y*, *Y* = {1, 2, …, *c*}, where *j* = 1, 2, …, *s*.

MCBFS (Fig. 10a, b) is a supervised learning method. *c* classes are taken as *c* known clusters, so the *i* th cluster center *v*_*i*_ = (*v*_i1_, *v*_i2_, …, *v*_*ip*_) can be calculated as follows:

1 $$ {v}_{ik}=\frac{\sum_{x_j\epsilon {c}_i}{x}_{jk}}{\left|{c}_i\right|} $$

where *i* = 1, 2, …, *c* − 1, *c*; *k* = 1, 2, …, *p* − 1, *p*; *j* = 1, 2, …, |*c*_*i*_| − 1, ∣ *c*_*i*_∣; ∣*c*_*i*_∣ represents the number of samples in cluster *c*_*i*_.

It is necessary to determine a better dissimilarity measure before clustering because different measures may affect the results of clustering. Chen et al. presented a KBCGS algorithm based on the Gaussian kernel measure and improved the classification performance on cancer gene expression data [63]. Wang et al. presented a SIMLR framework for analysis and visualization of single-cell data, which greatly enhanced clustering performance and interpretability via multi-kernel learning [36]. In the default implementation of MCBFS, we need to calculate the dissimilarity between expression values of gene and cluster center. Thus, to obtain a better dissimilarity measure, one possible method is to adjust the velocity of decrement in the range of distance between two values. In this work, multi-scale distance function with different parameters is designed to calculate the dissimilarity between the *k* th gene expression value of the *j* th sample and the *k* th dimension expression value of the *i* th center. The *n* th distance function takes the following form:

2 $$ {d}_n\left({x}_{jk},{v}_{ik}\right)=1-{e}^{-{\gamma}_n{\left({x}_{jk}-{v}_{ik}\right)}^2} $$

where *n* = 1, 2, …, *m*; where *m* is the number of distance functions; the parameters *γ*_*n*_ can be calculated as follows:

3 $$ {\gamma}_n=\frac{\sigma_n\times \left[{\left({u}_n\right)}_{max}+{\left({u}_n\right)}_{min}\right]}{2},{u}_n=\left({u}_{n1},{u}_{n2},\dots, {u}_{np}\right),{u}_{nk}={\left({x}_k\right)}_{max}-{\left({x}_k\right)}_{min} $$

where set different σ_*n*_ can produce different distance functions and *k* = 1, 2, …, *p*. *x*_*k*_ is a vector that consists of the *k* th gene expression value of samples.

In the process of clustering, we calculate individually each gene through each distance function. The general dissimilarity measure is obtained as sum of different distance results between sample and cluster center. Then the sample *x*_*j*_ and cluster centroid *v*_*i*_ can be calculated by multi-scale distance function as follows:

4 $$ d\left({x}_j,{v}_i\right)={\sum \limits}_{n=1}^m\left[{\sum \limits}_{k=1}^p{d}_n\left({x}_{jk},{v}_{ik}\right)\right] $$

In our method, we assume that the same gene has the same weight in all clusters (global adaptive distance), taking classes as known clusters. Different distance functions and genes are assigned different weights. To select genes that are more related to cancer and reduce the number of genes, we introduce the parameters *P*_*n*_ and *W*_*k*_ into the optimization function. Based on the clustering method, the objective of the MCBFS method is obtained by minimizing the following function:

5 $$ J=\sum \limits_{n=1}^m{P}_n\left[\sum \limits_{i=1}^c\sum \limits_{x_j\in {c}_i}\sum \limits_{k=1}^p{W}_k{d}_n\left({x}_{jk},{v}_{ik}\right)\right]+\updelta \sum \limits_{k=1}^p{W}_k^2+\rho \sum \limits_{n=1}^m{P}_n{logP}_n,s.t\;\left\{\begin{array}{c}{W}_k\in \left[0,1\right]\\ {}{\sum \limits}_{k=1}^p{W}_k=1\end{array}\right.,\left\{\begin{array}{c}{P}_n\in \left[0,1\right]\\ {}{\sum \limits}_{n=1}^m{P}_n=1\end{array}\right. $$

where *c* represents the number of categories; δ and *ρ* are non-negative tuning parameters; *P*_*n*_ represents the importance of the *n* th distance function for distinguishing tissue samples; *W*_*k*_ denotes the *k* th gene’s ability to distinguish tissue samples; *v*_*i*_ = (*v*_i1_, *v*_i2_, …, *v*_*ip*_) is the *i* th class center. The above optimization problem needs to solve four variables: *P*_*n*_, *W*_*k*_, δ and *ρ*. *P* = (*P*_1_, *P*_2_, …, *P*_*n*_, …, *P*_*m*_) and *W*= (*W*_1_, *W*_2_,…, *W*_*k*_, …, *W*_*p*_) are the coefficients to be estimated by optimizing the objective function (5), which represents the relative importance for classification of different distance functions and different genes, respectively.

The objective function has three terms in eq. (5). The first term in the formula enables us to obtain compact clusters. The second term represents the sum of the square of the gene weights. In the third term, the weight of the distance function is constrained to avoid selecting a single distance function. This regularization can improve the quality of clustering [36]. By combining three terms and choosing δ and ρ properly, the minimum value of the objective function, and optimal weight value of distance function and gene can be obtained.

To optimize equation (5) with respect to *W*_*k*_ and *P*_*n*_, we can obtain the following Lagrange function without constraint:

6 $$ J\left({P}_n,{W}_k,{\lambda}_1,{\lambda}_2\right)=\sum \limits_{n=1}^m{P}_n\left[\sum \limits_{i=1}^c\sum \limits_{x_j\in {c}_i}\sum \limits_{k=1}^p{W}_k{d}_n\left({x}_{jk},{v}_{ik}\right)\right]+\updelta \sum \limits_{k=1}^p{W}_k^2+\rho \sum \limits_{n=1}^m{P}_n{logP}_n+{\lambda}_1\left(\sum \limits_{k=1}^p{W}_k-1\right)+{\lambda}_2\left(\sum \limits_{n=1}^m{P}_n-1\right) $$

where *λ*_1_ and *λ*_2_ are the Lagrangian coefficients.

Using the Lagrange multiplier method, minimization of equation (6) with respect to *W*_*k*_ and *P*_*n*_, we obtained the following equations of variables:

7 $$ {W}_k=\frac{1}{p}+\frac{1}{2\delta}\left[\frac{\sum_{k=1}^p{\sum}_{n=1}^m{P}_n{A}_{nk}}{p}-\sum \limits_{n=1}^m{P}_n{A}_{nk}\right], $$

To conveniently describe equation (7), where

8 $$ {A}_{nk}=\sum \limits_{i=1}^c\sum \limits_{x_j\in {c}_i}{d}_n\left({x}_{jk},{v}_{ik}\right) $$

In addition, we can obtain the equation of *P*_*n*_:

9 $$ {P}_n=\frac{\exp \left(-\frac{1}{\rho }{\sum}_{i=1}^c{\sum}_{x_j\in {c}_i}{\sum}_{k=1}^p{W}_k{d}_n\left({x}_{jk},{v}_{ik}\right)-1\right)}{\sum_{n=1}^m\exp \left(-\frac{1}{\rho }{\sum}_{i=1}^c{\sum}_{x_j\in {c}_i}{\sum}_{k=1}^p{W}_k{d}_n\left({x}_{jk},{v}_{ik}\right)-1\right)} $$

Therefore, we can update *W*_*k*_ and *P*_*n*_ using equations (7) and (9). The greater the weight of genes, the greater the ability to provide information. Thus, top-ranked genes may be helpful for tumor classification. In equation (5), the choices of δ and *ρ* are important in the MCBFS algorithm. The values of δ and *ρ* should have the same order of magnitude as the first term when they are chosen [64]. We compute δ iteratively as follows:

10 $$ {\updelta}^{(t)}=\upalpha \times \frac{\sum_{n=1}^m{P}_n^{\left(t-1\right)}\left[{\sum}_{i=1}^c{\sum}_{x_j\in {c}_i}{\sum}_{k=1}^p{\mathrm{W}}_k^{\left(t-1\right)}{d}_n\left({x}_{jk},{v}_{ik}\right)\right]}{\sum_{k=1}^p{\left({\mathrm{W}}_k^{\left(t-1\right)}\right)}^2} $$

Similar to the derivation of equation (10), we can obtain ρ as follows:

11 $$ {\uprho}^{(t)}=\upbeta \times \frac{\sum_{n=1}^m{\mathrm{P}}_n^{\left(t-1\right)}\left[{\sum}_{i=1}^c{\sum}_{x_j\in {c}_i}{\sum}_{k=1}^p{\mathrm{W}}_k^{\left(t-1\right)}{d}_n\left({x}_{jk},{v}_{ik}\right)\right]}{\sum_{n=1}^m{\mathrm{P}}_n^{\left(t-1\right)}\mathit{\log}{\mathrm{P}}_n^{\left(t-1\right)}} $$

where α and β are non-negative tuning parameters.

In the experiment, we initialized the weight of distance functions and genes into uniform distribution vectors; i.e.

12 $$ P=\left(\frac{1}{m},\frac{1}{m},\dots, \frac{1}{m}\right),W=\left(\frac{1}{p},\frac{1}{p},\dots, \frac{1}{p}\right) $$

where *m* is an adjustable parameter and denotes the number of distance functions. The different scale values may influence the prediction performance. Wang et at [36]. proved that the clustering accuracy will increase as the number of kernels increase. If the number of kernels achieves a certain, the clustering accuracy will saturate. They set 55 different kernels and greatly enhanced clustering performance. In this work, we designed some comparison experiments to find a suitable value. The results have been shown in Figure S5. To save time and obtain better performance, *m* was set to 50. *p* denotes the number of genes. After repeated experiments, we took α = 0.5, β = 0.5. In the MCBFS algorithm, the maximum number of iterations was set to 100. The details of the workflow and iterative process are shown in Fig. 10a and b.

### Network analysis identified hub informative genes

In this work, we downloaded GSE10072 and GSE7670 lung adenocarcinoma data sets from the Gene Expression Omnibus (GEO) database. All genes of the two data sets were ranked by MCBFS, and 200 highly ranked genes were retained as candidate shared informative genes, respectively. Shared informative genes were screened using Venn analysis from candidate genes. To identify possible hub informative genes from shared informative genes, the hub proteins were identified from the complex protein-protein interaction (PPI) network formed by proteins encoded through shared informative genes.

The shared informative genes were uploaded in the Search Tool for the Retrieval of Interacting Genes database (STRING) (https://string-db.org) [65]. The PPI network data were downloaded by setting the minimum required interaction score at 0.400 and visualized by Cytoscape software. A plugin app Cytohubba [66] was used to provide aids for further screening of hub informative genes in the Cytoscape. Some different methods have been proposed to screen the key nodes in the network [20, 67]. The ensemble algorithm has demonstrated its effectiveness and potential [68]. To obtain the best integrated effect, we developed an ensemble algorithm that integrates 10 individual network recognition algorithms (including Degree, Maximal Clique Centrality, MNC, Closeness, BottleNeck, EcCentricity, Radiality, EPC, Betweenness and Stress) [66] and obtains the weighted average.

Finally, the top 10 genes were screened by the above network recognition ensemble algorithm as hub informative genes. They may be potential biomarkers and therapeutic targets for the precise treatment and diagnosis of diseases. In the clinical environment, this means that the diagnosis and prognosis of the diseases are possible, and the eventual treatment of the disease is clear.

### Wrapper identify biomarkers

Biomarkers should have higher sensitivity and specificity, be good for classification and have an important influence on the development and occurrence of the diseases at the same time. The Kaplan Meier plotter (www.kmplot.com) [69], an online database, was used to evaluate the prognostic value of 10 hub informative genes. Specifically, genes with logrank *P* value less than 0.05 were screened out as key informative genes of tumor by survival analysis in hub informative genes.

In this work, to obtain a better classifier, two popular classifiers were used to obtain the average classification error rate by performing 10-fold cross-validation on all data sets. Figure 1 demonstrates that the kNN classifier is potentially better for small gene set classification problems after feature selection. To research the possibility of these genes as biomarkers of identifying the occurrence of tumors, the kNN classifier was combined as a wrapper to identify the classification capability of genes and simple gene combinations. We obtained a small number of key informative genes (no more than 10 genes) by survival analysis. Since the number of our key informative genes set is small, exhaustive best subset search (ES) [70] was used as the feature search algorithm to find a small subset of genes that could ensure highly reliable classification. The results were obtained by a new data set as an independent test set for correcting the selection bias to obtain a more reasonable result for the proposed method and further explore the key informative genes. In the application process of McbfsNW framework, the combination of two data sets (GSE10072 and GSE7670) served as the training set, and the new data set (GSE43458) served as an independent testing set. First, we classified the test set with only one gene. Then, we repeat this process with all possible 2-gene combinations in the key informative genes, and so on. The results shown that it is possible to construct prediction rules from only a few genes, and the prediction error rate can be negligible.

## Supplementary information

**Additional file 1 :Figure S1.** (A). Normal t-score quantile plot of GSE10072 data set. (B). Normal t-score quantile plot of GSE43458 data set. **Figure S2.** Venn diagram of informative genes from two data sets (GSE10072, GSE7670). **Figure S3.** The protein-protein interaction network diagram of shared informative genes. **Figure S4.** Survival analysis of 10 hub genes. **Figure S5.** The relationship between Acc value and parameter *m*.

## Data Availability

The lung adenocarcinoma data sets (GSE10072, GSE7670 and GSE43458) analysed in the work can be available at the Gene Expression Omnibus (GEO) database (https://www.ncbi.nlm.nih.gov/geo/). The datasets of Table 1 used can be available from the corresponding author on reasonable request. The seven benchmark feature selection algorithms can be available at the website: http://featureselection.asu.edu/old/software.php. In terms of the six state-of-the-art supervised feature selection methods, the codes of SDSPCA and DKBCGS method can be obtained from the corresponding author of references; The ILFS and infFS methods can be available at: Giorgio (2020), Feature Selection Library (https://www.mathworks.com/matlabcentral/fileexchange/56937-feature-selection-library); The SPEC method can be available at the website: http://featureselection.asu.edu/old/software.php; The Supervised Multi-Cluster Feature Selection method can be available at: https://github.com/ZJULearning/MatlabFunc/tree/master/FeatureSelection.

## Abbreviations

MCBFS: Multi-scale supervised clustering-based feature selection
MCBFSnw: MCBFS algorithm with network recognition ensemble algorithm and feature selection wrapper
SDSPCA: Supervised discriminative sparse PCA
GAN: Generative adversarial networks
LUAD: Lung adenocarcinoma
NSCLC: Non-small cell lung cancer
SCLC: Small cell lung cancer
Kappa: Cohen’s Kappa coefficient
BID: BI-D1870
STRING: Search Tool for the Retrieval of Interacting Genes
ES: Exhaustive best subset search
