# Supplementary material for: Multi-scale supervised clustering-based feature selection for tumor classification and identification of biomarkers and targets on genomic data
Source: BMC Genomics. 2020 Sep 22;21:650. doi: 10.1186/s12864-020-07038-3 (PMC7510277; doi:10.1186/s12864-020-07038-3)
Supplement: Supplementary file 1 — Additional file 1 :Figure S1. (A). Normal t-score quantile plot of GSE10072 data set. (B). Normal t-score quantile plot of GSE43458 data set. Figure S2. Venn diagram of informative genes from two data sets (GSE10072, GSE7670). Figure S3. The protein-protein interaction network diagram of shared informative genes. Figure S4. Survival analysis of 10 hub genes. Figure S5. The relationship between Acc value and parameter m. [file 12864_2020_7038_MOESM1_ESM.docx]

**Figure S1**

**
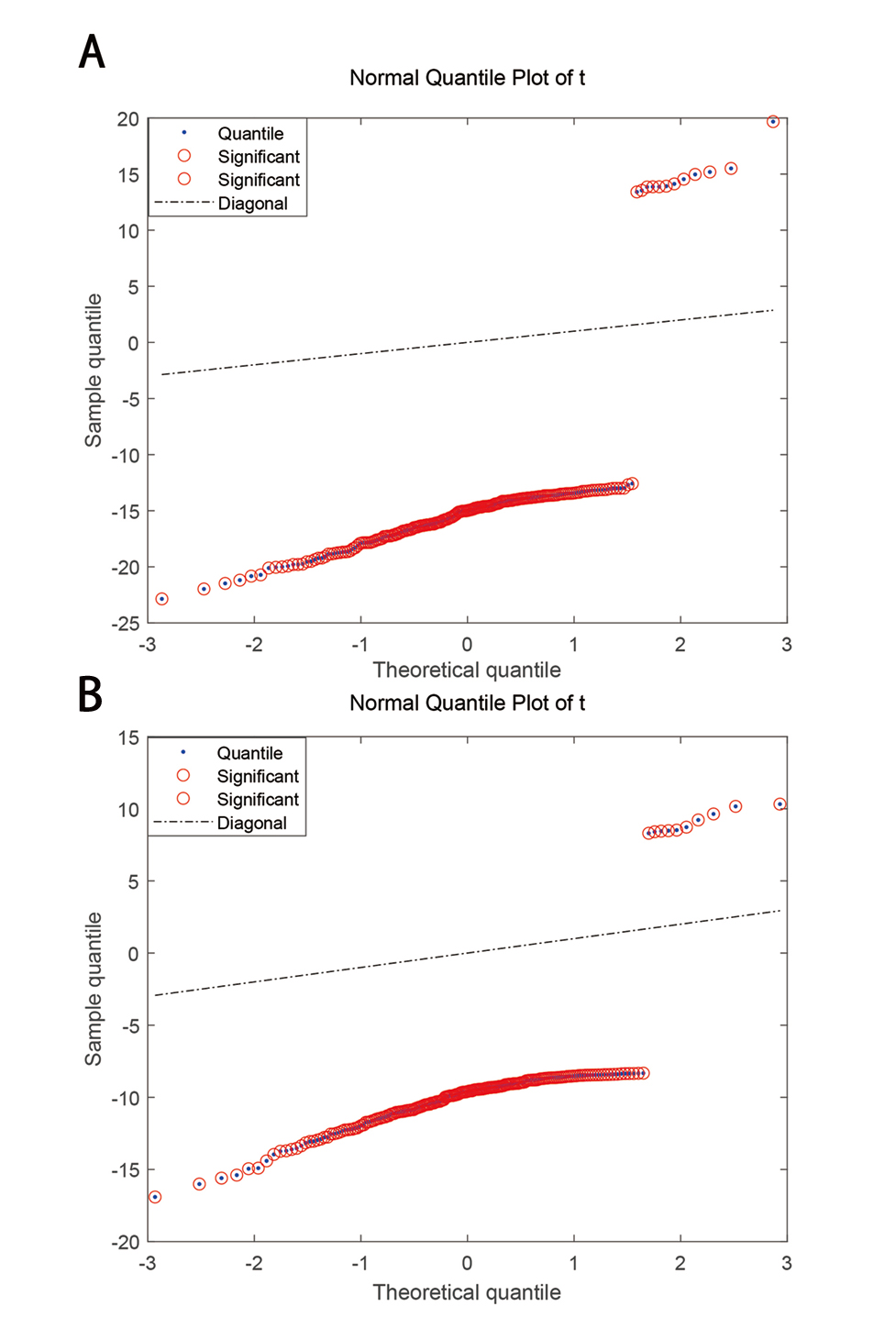
**

**Figure S2**

**
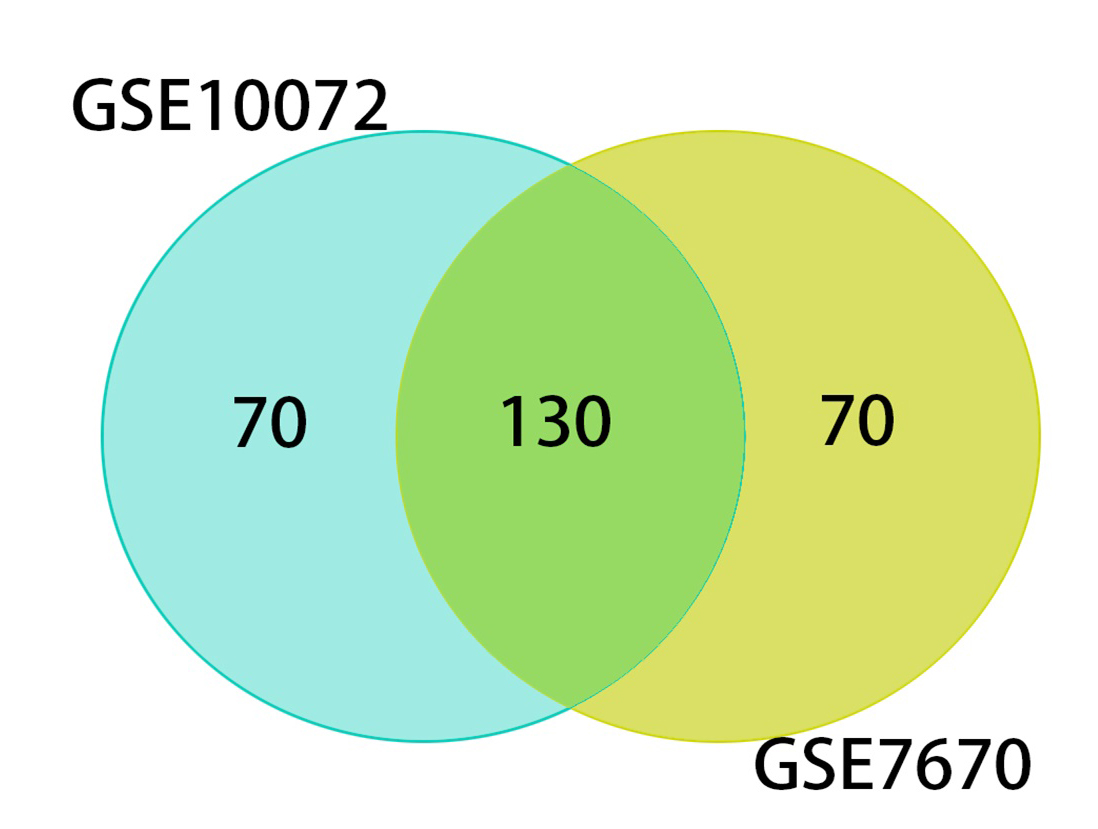
**

**Figure S3**

**
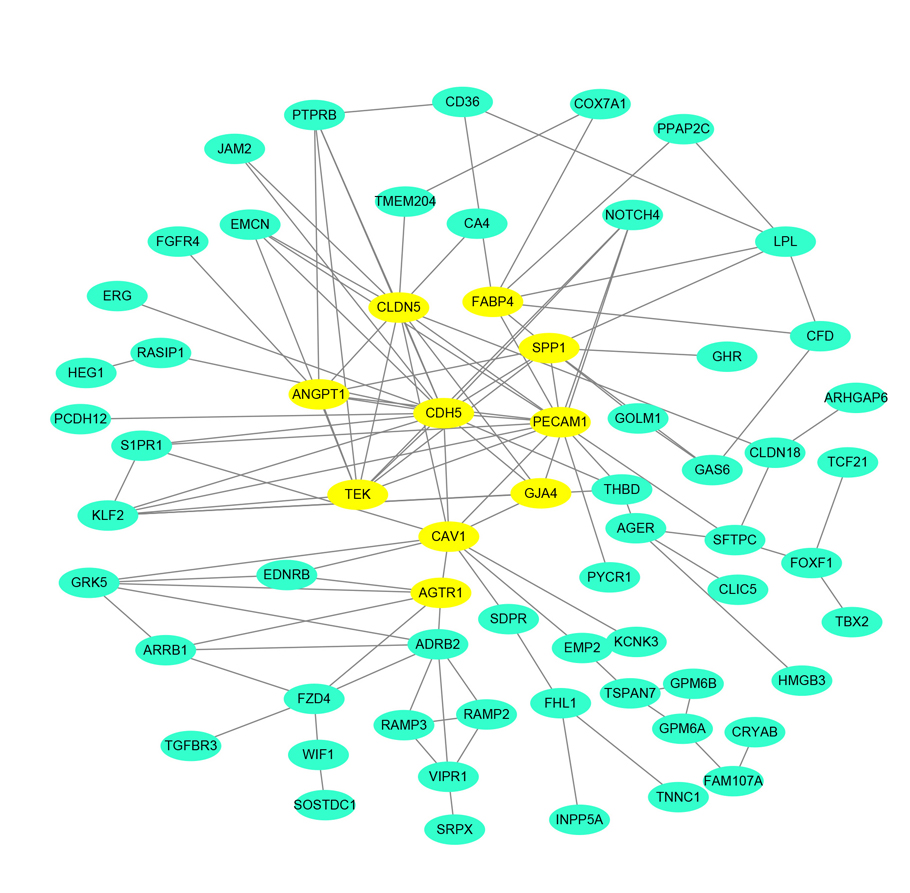
**

**Figure S4**

**
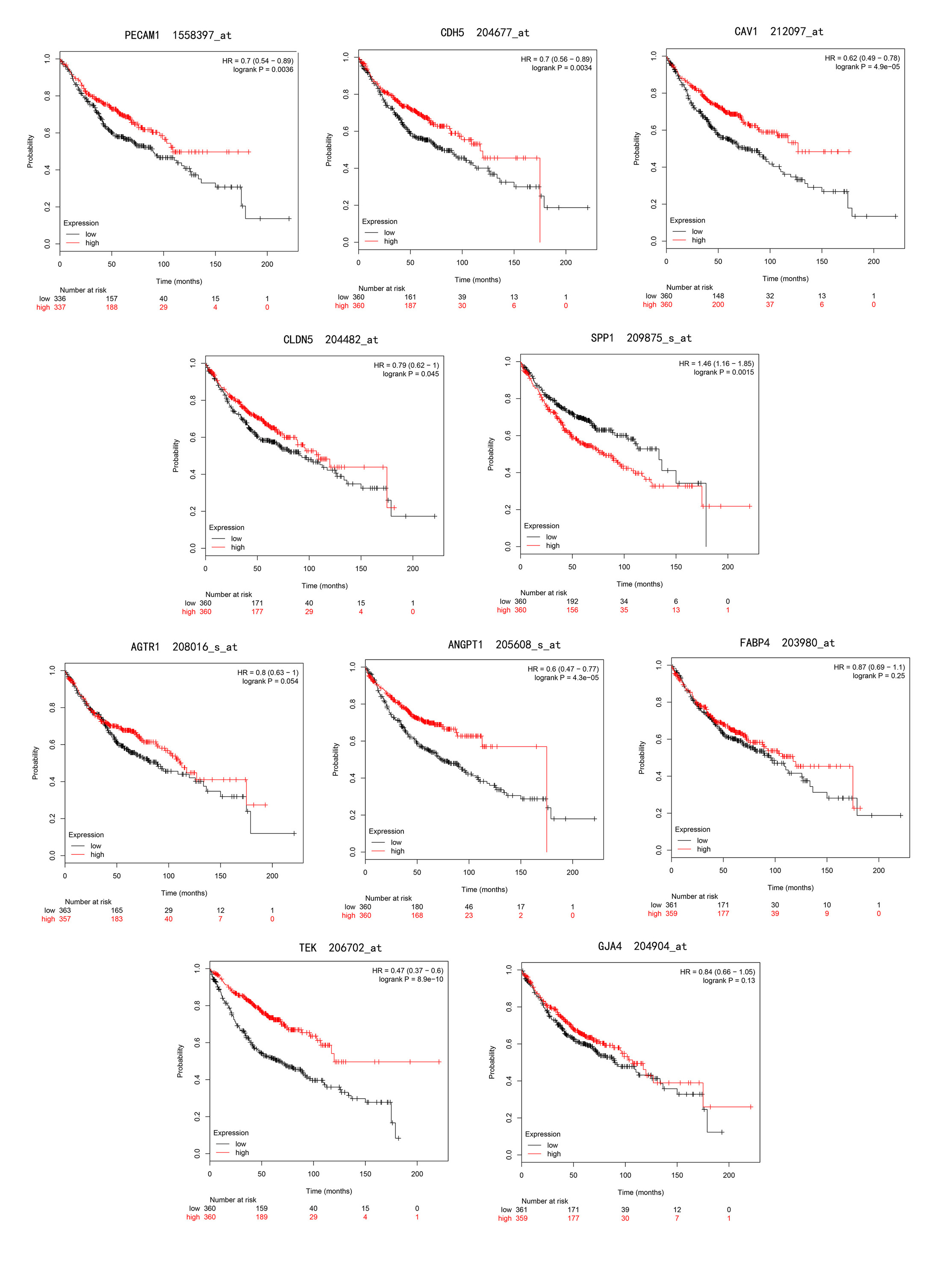
**

**
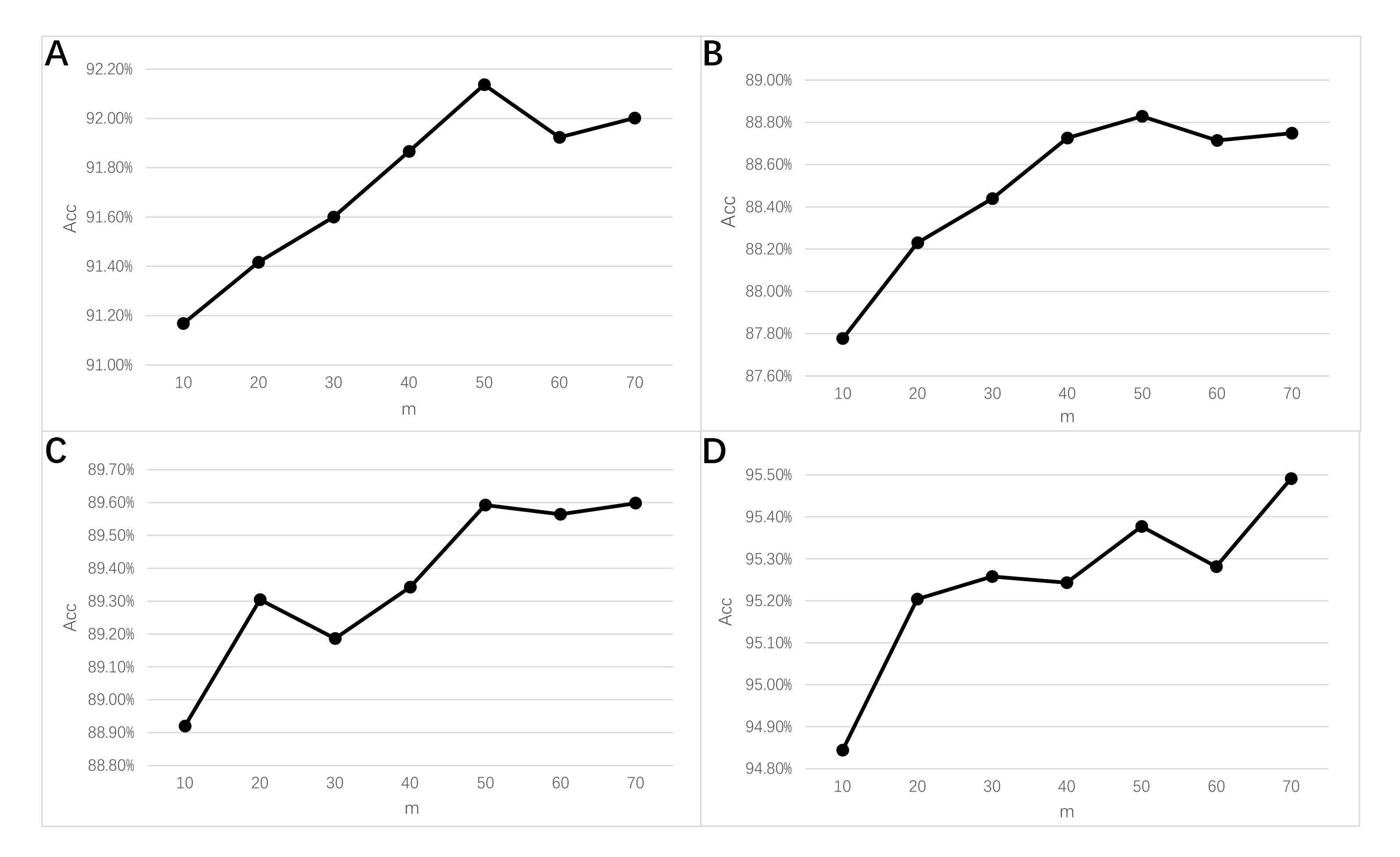
**

**Figure S5. The relationship between Acc value and parameter** $\boldsymbol{m}$ **of the MCBFS.** (A) The relationship between average Acc value and parameter $m$ of four two-class data sets with SVM classifier. (B) The relationship between average Acc value and parameter $m$ of four two-class data sets with kNN classifier. (C) The relationship between average Acc value and parameter $m$ of four multi-class data sets with SVM classifier. (D) The relationship between average Acc value and parameter $m$ of four multi-class data sets with kNN classifier.

The performances of the multi-scale distance method yield better than the single distance method, so the range of $m$ is from 10 to 70 and each step is 10. We performed 10-fold cross-validation for 10 times to obtain a reliable predictive performance on the types of two-class cancer data sets and multi-class cancer data sets by SVM and kNN classifiers, respectively. From the figure, the average Acc is enhanced when $m$ increases from 10 to 50, indicating the multi-scale distance function provides effective information. If the number of $m$ achieves a certain, the Acc will saturate. To save time and obtain better performance, $m$ was set to 50.
